# Socioeconomic Status and Vulnerability to HIV Infection in Uganda: Evidence from Multilevel Modelling of AIDS Indicator Survey Data

**DOI:** 10.1155/2018/7812146

**Published:** 2018-06-07

**Authors:** Patrick Igulot, Monica A. Magadi

**Affiliations:** ^1^University of Sunderland in London, UK; ^2^University of Hull, UK

## Abstract

**Background:**

There is controversy on the association between socioeconomic status (SES) and HIV infection. Some evidence claims higher SES is negatively associated with HIV infection while others report the reverse.

**Objectives:**

To examine the association between SES and HIV infection in Uganda and to examine whether the SES-HIV relationship varies by gender, rural-urban place of residence, and time (2004-2005 and 2011) in Uganda.

**Methods:**

Multilevel analysis was applied to 39,766 individual cases obtained in 887 clusters of Uganda HIV/AIDS Indicators Survey conducted in 2004-2005 and 2011.

**Results:**

Household wealth is associated with increased vulnerability in the general population and in rural areas. Compared with no educational attainment, secondary or higher education is associated with reduced vulnerability to the risk of HIV infection by 37% in the general population. However, this effect was stronger in urban than rural areas. Besides individual-level factors, unobserved community factors too play an important role and account for 9% of unexplained variance after individual-level factors are considered.

**Conclusion:**

Household wealth increases vulnerability but education reduces it. The social environment influences vulnerability to HIV infection independent of individual-level factors. HIV/AIDS awareness targeting sexual practices of wealthy individuals and those with primary-level educational attainment together with improving educational attainment and addressing contextual factors influencing vulnerability to HIV infection are necessary strategies to reduce HIV infections in Uganda.

## 1. Introduction

The association between socioeconomic status (SES) and HIV infection in sub-Saharan Africa (SSA) is controversial. Considerable research attention has been given to the relationship between SES and HIV in SSA, a region that suffers a disproportionate higher burden of HIV/AIDS. Some studies suggest that people with low, while others suggest that those with high SES are more vulnerable to HIV infection [1–3]. More studies have demonstrated the positive relationship between SES and vulnerability to HIV infection in SSA [4–6]. Previous studies have used diverse measures of SES, including employment. However, in this paper, wealth and education are used as the main measures of SES because they are consistently defined and measured in the AIDS Indicator Survey (AIS).

### 1.1. Household Wealth Status

Wealth status is linked to HIV infection through complex pathways. The first link is through the income effect [7] that may be in the opposite direction. People with high income tend to lead lifestyles associated with increased number of sexual partners which increases their vulnerability to HIV, while those with low income may be unable to access HIV services also leading to increased vulnerability [4, 6, 7]. Poverty makes people vulnerable to HIV in diverse ways including dropping out of school; marrying early; loss of livelihood; and being homeless due to displacement by war, all of which have been linked to increased HIV vulnerability (e.g., [4, 8]).

### 1.2. Educational Attainment

The education-HIV evidence is also mixed. Some studies indicate that education is negatively associated with HIV infection [9, 10] while others report a positive association [1, 11–14]. There is more convincing argument in support of the former; for example, education may be associated with HIV infection through schooling. Schooling keeps young people away from environments which would increase their vulnerability to HIV infection and inspires students to develop long term goals. These contribute to delaying sex, which makes young people avoid HIV infection [15, 16]. Higher educational attainment (defined here as complete secondary or higher education) provides knowledge, which individuals use to avoid HIV infection [6, 17], and provides employment, which enhances the capacity of people to act on their plans to reduce vulnerability [4, 15, 16].

The SES-HIV evidence is controversial and context specific. However, one study [2] examined changes in HIV prevalence over time, and as much as the role of rural-urban residential area and gender in the construction of vulnerability has been addressed in the previous research (e.g., [14, 18, 19]) there is none about Uganda. Besides the controversy on SES, majority of previous research has focused on the influence of individual characteristics (demographic factors) on their risk of being infected with HIV [20–23]. Scholars, including Clarke et al. [24], argue that the personal characteristics of the individual do not fully explain their risk of HIV infection. In this research, we use multilevel modelling, a method that simultaneously measures the effect of individual characteristics (observed effects) and the effects of community characteristics (unobserved effects) [24, 25]. Understanding social context is important because these factors cause social practices [26]; they increase vulnerability to HIV infection and curtail the success of proven HIV/AIDS interventions (e.g., [27, 28]), while others protect individuals from HIV risk.

This study contributes to these debates by providing evidence from Uganda, a country which was previously a global model in the response to HIV/AIDS [29] but where prevalence has been rising from 6.2% in early 2000s, to 6.4% in the 2005 to 7.3% in 2012 [30]. As much as this may be due to increased survival rates because of antiretroviral treatment [31–33] or other remedies [34], this worrying trend in HIV/AIDS epidemic has occurred in the context of grim socioeconomic development, with 65 percent of the population being classified as being poor [35, 36] and the majority having only primary or no education [37].

The objectives of this research are as follows:

1. To examine the association between socioeconomic status and HIV infection in Uganda.
2. To examine whether the SES-HIV relationship varies by gender, rural-urban place of residence, and time (2004-5 and 2011) in Uganda.

## 2. Data and Methods of Analysis

### 2.1. The Data

This research is based on a nationally representative sample of 22,979 women and 18,418 men of reproductive age from 20,869 households with 33,692 rural and 7,705 urban respondents obtained from the 2004-2005 and 2011 Uganda AIS. AIDS Indicator Survey is a global research programme that collects data to inform national policies and facilitate international comparison [38]. All adults aged 15–59 years interviewed were also requested to voluntarily give blood for HIV testing. A two-stage sampling strategy was applied, starting with the selection of clusters in each region and then sampling 25 households in every cluster [39]. In this research, a cluster is synonymous with a community, which is defined as 50–500 households clustered within the same geographical area. This classification is based on the country's census sampling frame, which classifies these households into primary sampling units (i.e., clusters) or communities. This is the standard definition of community in the Demographic and Health Survey (DHS) data for measuring community effects [40].

The survey protocols were approved by the Science and Ethics Committee of Uganda Virus Research Institute, ICF Macro Institutional Review Board, Ethics Review Committee at CDC, Atlanta, and the Ethics Committee of Uganda Council for Science and Technology [38].

### 2.2. Sample Characteristics

This research is based on 39,766 individual cases aged 15–49 years (women) and 15–59 (men). Sixty-five percent of the respondents were under 35 years of age with an average age of 30.5 years for females and males. There were 82.7% rural residents in the surveys, with the remaining 17.3% being urban residents. Women respondents comprised 55.6% of the sample. Most respondents did not have secondary or higher education. Respondents with primary or no educational attainment comprised 72.9%; more women had less education than men. Among rural residents, 89.6% had primary or no education at all. The mean years of education for men and women were 5.7. These sample characteristics reflect the structure of the general Ugandan population (e.g., [37]).

### 2.3. Methods of Analysis

Multilevel logistic regression was used to predict the probability of having HIV infection, represented by 1, against the probability of not having, which was represented by 0 [5]. To create the wealth index, DHS uses a subset of indicators common to rural and urban areas to create wealth scores for households in both areas. Some of the indicators used are those that measure access to the mass media, access to efficient means of communication, household's capacity to afford pricy items and to hygienically store food, and access to public services, markets, and exposure to other development areas. Other indicators used are those that measure access of households to means of production and social standing seen through characteristics of the dwelling (floor, wall, and roofing materials used), type of drinking water source, type of energy used, and type of toilet facilities that a household uses.

In the second step, separate component scores are produced for households in urban and rural areas using area-specific indicators. The third step entails combining the separate area-specific scores to produce a nationally applicable combined wealth index, by adjusting the area-specific score through regression on the common component score [41, 42]. Because of the compositional nature of the wealth index and difference in country contexts, we measure SES using this survey/country specific wealth/poverty index [42] classified into five quintiles: lowest; second; middle; fourth; and highest [41]. Educational attainment is categorized into five ordinal levels: no education; incomplete primary; complete primary; incomplete secondary; and complete secondary and higher education.

Analysis started with bivariate analysis of HIV status by wealth and education. We control for SES factors first for 2 main reasons: first, as an independent variable(s) of interest, we want to know the size of its effect; and second, given its known correlation with many other variables, controlling for SES first allows us to know its contribution to the overall model. This was then extended to multivariate analysis which used the logit model to sequentially fit two-level nested models. The analysis first controlled for potential confounders before adding sexual behavior factors in the final model to identify potential pathways through which SES is associated with HIV prevalence.

During modelling, analysis started by obtaining general models based on pooled data. After analysis of pooled data, interactions between SES and gender, rural-urban residence, and time were obtained to explore potential variations in the effect of SES by these factors. This was necessary to investigate whether evidence of heterogeneity in the SES-HIV relationship regarding gender and urban/rural residence that has been observed in other SSA settings [19, 43–45] is applicable in Uganda and to explore whether the nature of the relationship has changed over time. Every step started by running a model with wealth and education only and then adding other covariates to establish potential pathways of the link between SES and HIV vulnerability, using MLwiN [46].

This research focuses on gender and rural-urban residential areas because these concepts are important dimensions of SES and are significant in the construction of HIV vulnerability and yet they are less explored in SSA [5]. This analysis focused on residential areas because they represent physical spaces and networks for the spread of HIV and present different conditions with influence on HIV vulnerability [7]. Change in vulnerability to risk over 2 time periods, i.e., 2004-2005 and 2011, was considered important given its relevance to changes in policies and programmes [47] which are likely to influence HIV vulnerability.

## 3. Descriptive Findings

### 3.1. HIV Prevalence by Sample Characteristics

The overall prevalence of HIV was 6.4% in 2004-2005 but it increased to 7.3% in 2011 and was higher among women than in men (Table 1). With respect to SES, there was no evidence of a significant association between wealth status and HIV prevalence for both males and females in 2004-2005 and 2011. For education, men with no educational attainment had the highest prevalence in 2004-2005 and 2011. It is interesting to note that in 2004-2005 while men with the highest educational attainment had the lowest HIV prevalence, for women, HIV prevalence was lowest among those with no education. In 2011, there was evidence of higher educational attainment beyond primary level being associated with reduced HIV prevalence for both males and females.

Interesting patterns in HIV prevalence were observed by other key factors, besides SES. The overall rise in HIV prevalence between 2004-2005 and 2011 was mainly driven by a rise in prevalence among older men and women aged 35 years and above, rural residents, and never married men. HIV prevalence was particularly high among widowed men in 2004-2005 at 27%. In terms of age, HIV prevalence in 2004-2005 was highest among women aged 25–34 years and men aged 35–44 years. In 2011, prevalence was highest among both men and women aged 35–44. The HIV prevalence among women in female headed households is about twice that in male headed households in 2004-2005 and 2011. However, prevalence among women in male headed households was lower than men in the same household. These patterns suggest that the observed higher prevalence among women than men is driven by heightened vulnerability among women in women headed households.

**Figure 1:**
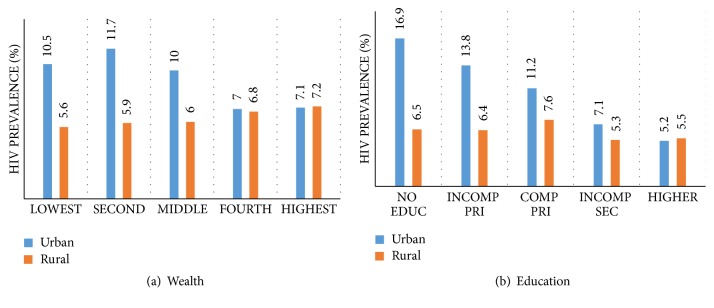
HIV prevalence by household wealth status (a) and educational attainment (b) in rural and urban areas, UAIS, 2004-2005 and 2011 (*n* = 39,767).

Figure 1 shows further how HIV prevalence varies by SES characteristics and by urban/rural residence. In Figure 1(a), there is a positive wealth-HIV gradient in rural areas, steadily rising from a prevalence of 5.6% among the lowest to 7.1% among the highest. However, in the urban areas, the trend is reversed. HIV prevalence declines from 11.7% among people in the second wealth category to 7.2% among those in the highest. In Figure 1(b), education is negatively associated with HIV in urban areas but in rural areas, a negative but somewhat consistent trend can be observed only after complete primary education.

## 4. Multilevel Findings

In the multilevel modelling, four models were fitted, starting with wealth and education in the first model and then controlling for other characteristics. In the final model, evidence in Table 2 shows that there is a general positive association between wealth and HIV prevalence, with prevalence in the highest wealth households being 20% higher than in the lowest wealth quintile households after controlling for background characteristics. The higher HIV prevalence among those in the highest wealth quintile becomes evident when socioeconomic and demographic factors were controlled for in Model 3. However, this ceased to be significant in Model 4 when sexual behavior factors were controlled for, suggesting that the higher HIV vulnerability among wealthy individuals was partly explained by sexual behavior factors, which is consistent with existing literature.

For education, secondary or higher educational attainment was associated with reduced odds of HIV infection, while primary education was associated with increased odds, compared to no education. However, the higher HIV vulnerability associated with primary-level education only became apparent in Model 3 when background socioeconomic and demographic factors, especially, current marital status, were controlled for and ceased to be significant when sexual behavior factors were controlled for. Overall, complete secondary education was associated with lower HIV prevalence, conditional on background characteristics and sexual behaviors. For instance, if those with complete secondary or higher education had the same background characteristics and sexual behavior (to the extent measured in our model) as those with no education, their HIV prevalence would still be 37% lower.

To check the robustness of the findings in this research, we rerun the analysis that controlled for age in single-year dummies in Models 3 and 4 instead of 10-year age categories/dummies (Table 3). Using 39,766 cases used to produce results in Table 2 with age of 59 years having the least number of cases (203), we wanted to find out if the SES-HIV association would substantially change with this approach. The results in Table 3 did not show any substantial difference with those in Table 2 that are based on the analysis that controlled for age in broad 10-year age group categories.

### 4.1. Determinants of HIV Vulnerability by Urban/Rural Residence

The SES determinants of HIV prevalence in Uganda by urban/rural residence (Table 4) were further explored. HIV prevalence was negatively associated with incomplete secondary and complete secondary and higher educational attainment in urban areas. People with incomplete secondary were 36% less likely to be infected while those with complete secondary or higher were 57% less likely to have HIV compared to people with no education in urban areas with similar other characteristics. There was no evidence of a significant education effect in rural areas once sociodemographic factors and sexual practices were controlled for in the final model; when age was controlled for, the effects of higher education reduced from odds ratio 0.63 [0.46–0.87] to 0.93 [0.67–1.29] and when marital status was controlled for, the effects reduced further to 0.96 [0.79–1.17]. This indicates that these factors, especially age, confound the association between education and HIV vulnerability in rural areas.

Compared to men with the same other social characteristics, women were significantly more likely to be HIV-positive than men (average OR = 1.61 in rural and 2.13 in urban areas), suggesting that all other factors being equal, women were more vulnerable than men and the gender gap was greater in urban than rural areas. In relation to trends, there was evidence of significantly higher HIV prevalence in 2011 than 2004-2005 and in rural but not in urban areas.

### 4.2. Interaction Effects

To check the robustness of our results further, we investigated whether there were significant differences in association between SES and HIV prevalence by gender, rural-urban residential area, and across 2 surveys, 2004-2005 and 2011, interaction modelling was performed [48]. Wealth and education were separately interacted with residential area, gender, and time. There was no evidence of significant interactions between wealth status and rural/rural residence, gender, or time. However, secondary or higher education was associated with increased odds of being HIV-positive among rural residents (Table 5). This may point to the possible influence of social and sexual practices in increasing vulnerability among people with higher educational attainment in rural areas.

### 4.3. Effect of Unobserved Community-Level Factors

Random effects were used to calculate the effects of community-level factors on HIV vulnerability based on the following formula [49].

(1) $$\begin{array}{c} p=\frac{uoj}{\left(uoj+eij\right)}, \end{array}$$

where *p* is the intracluster correlation, *eij* is variation at level 1 (individual), which is represented by 3.29 [46, 49] (for Logistic Regression) and *uoj* is variation at level 2 (Cluster). Community random variance estimates were used to calculate the intracluster correlation coefficient. Overall, after accounting for individual-level factors included in the model, 9% of the residual variance occurs at the cluster level. This community effect is notably stronger in rural (ICC = 0.13) than in urban (ICC = 0.05) areas.

## 5. Discussion

### 5.1. Household Wealth Status

There is an association between wealth status and HIV infection in the pooled 2004-2005 and 2011 data (Table 2), and in rural areas (Table 4). These findings were consistent with a previous Ugandan research [2] and those of other previous researches (e.g., [50]) showing higher HIV prevalence among individuals in wealthy households, especially rural residents. Interactions between wealth and gender, residential areas, and across two surveys (2004-2005 and 2011) were also examined but did not find any significant interactions.

The association between household wealth status and HIV infection was explained by current marital status. For example, when education, ethnicity, rural-urban place of residence, time (2004-2005 and 2011), and age were controlled for, the odds ratio for the highest wealth quintile was not significant. However, when current marital status was considered, the odds ratio for the highest wealth quintile increased to 1.21 [1.03–1.42], suggesting marital practices of wealthy people influence their vulnerability to HIV infection. This is consistent with the literature showing that relatively rich people or those living in wealthy households and having a higher rate of partner change, a phenomenon associated with relative autonomy and mobility, increased the vulnerability of such individuals to HIV infection [4, 5, 51].

Patterns from bivariate analysis provide no evidence of a significant association between wealth and HIV infection (Table 1), but the analysis by urban rural residence (Figure 1) suggests a positive gradient in rural areas and a negative one in urban areas. Although there was no evidence that the effect of wealth on vulnerability to HIV infection varied significantly by time, gender, or urban/rural residence based on interaction effects (Table 5), the analysis by urban/rural residence (Table 4) provides evidence of a positive association between wealth and HIV prevalence only in rural areas, consistent with patterns observed in other SSA settings (e.g., [44, 45]).

The positive wealth-HIV gradient observed in this research supports recent evidence by (e.g., [50]) using DHS data from 8 sub-Saharan African countries demonstrating wealth as an important driver of HIV infections in SSA. This reenforcement of evidence necessitates a shift in thinking and action aimed at preventing HIV infections. It is important to create HIV/AIDS awareness based on identified social and sexual practices that increase the vulnerability of wealthy people or those who live in wealthy households to HIV infections. Strategies for creating awareness targeting marital practices, multiple sexual partnership practices of, alcoholism, among other practices, by wealthy people, may specifically need to be prioritized. Nevertheless, it is important to recognize that since HIV prevalence depends on both HIV incidence and survival rates of those infected, the higher prevalence among those who are wealthier could be partly attributable to them living longer with the condition.

### 5.2. Educational Attainment

Higher educational attainment was consistently negatively associated with HIV prevalence. Overall, people with higher education were 37% less likely to be HIV-positive compared to those of similar other characteristics with no education. The education effect was stronger for those in urban than rural areas (Table 4). These findings (i.e., that people who had higher education were generally less likely to be infected) are in line with previous research (e.g., [9, 10, 48, 52, 53]).

The protective benefits of educational attainment were greater for people in urban areas than those in rural areas which had nonsignificant findings (Table 4). The greater benefit urbanites derive from education may be due to several factors. Kelly has suggested that education reduces vulnerability to HIV infection by enhancing self-protection, fostering the development of a value system, and adoption and promotion of behaviors that lower vulnerability to HIV risk [54]. For gender, the effect of educational attainment in reducing vulnerability was not significantly different between women and men (interaction effect not significant) once other significant factors were controlled for, but bivariate analysis showed interesting patterns. While men with secondary or higher education had the lowest HIV prevalence, for women, HIV prevalence was lowest among those with no education in 2004-2005 (Table 1). The apparent edge educated men enjoy over women is possibly due to their better access to other resources resulting from their “privileged” status in society [5].

### 5.3. Community Effects

The 9% residual variance apportioned to the community-level could be due to several factors. First, the improved road transport networks and the development of trading centres or urbanization. Proximity to a centre of development has been linked to increased vulnerability to HIV infection in SSA [18, 20, 55]. The community effect was observed to be stronger in rural than urban areas, consistent with patterns observed elsewhere [45]. In Uganda, rural residents with higher SES are more likely to frequent trading centres (periurban areas) to drink alcohol, to shop, and generally to socialize.

The unobserved community-level effects on vulnerability to HIV infection implies that, besides individual and household level characteristics, contextual factors also influence vulnerability to HIV infection (e.g., [20, 21]). It is thus pertinent that efforts to prevent HIV focus on the wider contextual factors that shape household factors and individual-level practices (e.g., [22, 23]). Community factors such cultural practices, health infrastructure, poverty and wealth, education, sexual violence and gender issues, and legal-policy and other institutional issues merit attention in this regard.

## 6. Conclusion

This research set out to examine the association between SES and HIV infection in Uganda. This research found significant positive associations between household wealth status and HIV infection at the population level, in rural areas, and among women. However, higher educational attainment was negatively associated with being infected with HIV at the population level, among women and men, in urban areas, and in 2011. Overall, secondary or higher education was associated with reduced vulnerability to HIV infection by 37%, compared to no education.

The second objective was to examine whether the SES-HIV relationship varies by gender, rural-urban place of residence, and time (2004-5 and 2011) in Uganda. This research found no evidence of significant interaction between wealth and gender and rural/urban residence. There was also no significant interaction between wealth and time in 2004-2005 and 2011. For education, there was a significant interaction with rural/urban residence which showed that secondary or higher education was less protective among rural compared to urban residents.

The general lessons from this study with respect to the relationship between wealth and HIV prevalence relate to the complex and context specific nature of the relationship. While studies elsewhere in sub-Saharan Africa have highlighted clear gender differences, suggesting increased vulnerability of poor women, the analysis for Uganda presented here provides no evidence of significant gender differences in the relationship between SES and HIV prevalence. This may be attributable to the fact that some of the factors responsible for an apparent relationship are already considered (e.g., increased vulnerability among women in women headed households accounting for the disadvantaged socioeconomic status of women) or may indeed reflect the context specific nature of the relationship.

The research also examined the effect of community-level factors on vulnerability to HIV infection. This research found that 9% of the residual variance could be attributable to the community environment, with the effect being substantially higher in rural than urban areas. About 5% of the total unexplained variance in HIV infection in urban areas was attributable to unobserved community-level factors, compared to 13% of unexplained variance in rural areas.

These findings call for policies to support people to attain higher education as a strategy to effectively prevent HIV infections; attainment of secondary or higher education provides prospects for multiple benefits ranging from knowledge, attitudes and capabilities, aspects that are transformable into economic resources. Education is also an avenue to achieve general social transformation that would influence a range of development aspects linked to health. Focusing attention on Uganda's education is particularly urgent given astounding evidence showing its gradual decline at primary level [56, 57]. Development attention also needs to be paid to the rural social environments, areas which substantially account for the greatest social influence in the construction of HIV vulnerability and which account for the increasing HIV prevalence in Uganda.

Further, these findings show that there are factors at the community level that influence vulnerability to the risk of HIV infection, evidence which justifies a focus of interventions on both individual and community factors such as sexual relationships, cultural norms, beliefs, and attitudes, socioeconomic status, availability of good health services, and religious values, among others. The findings validate and reenforce the need for community-level programme interventions including community HIV/AIDS education and making health services including those for HIV/AIDS accessible. Given the limited success of individually oriented HIV programme interventions in preventing HIV infection, an appreciation of the need to change approach is overdue.

These findings need to be interpreted bearing in mind the fact these were cross-sectional surveys which make it inappropriate to infer causality. This is because these surveys use prevalence estimates which do not reveal whether observed associations existed before infection or infection preceded the observations. Since antiretroviral therapy became widely available in SSA, people living with HIV can live longer for up to 10 or more years following the progression of HIV to AIDS (e.g., [33]). The increased survival rates and decreased mortality rates have thus contributed to increased HIV prevalence [31–33, 58].

Nevertheless, by testing respondents for HIV, these surveys establish objective measures of HIV prevalence which allow for an accurate measurement of associations with HIV. Further, higher HIV prevalence among people in wealthy households could be due to longer survival, a phenomenon known to influence prevalence. This research used data from Uganda's only 2 AIS, which limited the ability of the research to determine the trend of these associations over a longer period. Future research needs to track these trends over a longer period. As much as associations based on residential areas were observed, there was limited data to determine spatial distribution of cases, an aspect which future research also needs to address.

Notwithstanding the above limitations, this research, which was part of a bigger research for PhD in Sociology [59], supports research evidence reported elsewhere in SSA and it is the first multilevel modelling to demonstrate the effect of community factors and vulnerability to HIV infection in Uganda. Besides individual and household factors, community factors also influence vulnerability to the risk of HIV in Uganda. HIV/AIDS awareness targeting sexual practices of individuals in wealthy households and those with primary educational attainment together with improving educational attainment in Uganda and addressing contextual factors influencing vulnerability to HIV infection are necessary strategies to prevent HIV infections in Uganda. Given that these surveys are nationally representative; these findings are generalizable to the whole of Uganda and perhaps beyond.

## Figures and Tables

**Table 1:** Weighted HIV prevalence by key characteristics, Uganda AIDS Indicator Survey (UAIS), 2004-2005 and 2011.

Characteristic	UAIS, 2004-5				UAIS, 2011			
	Women		Men		Women		Men	
	% HIV+	Cases	% HIV+	Cases	% HIV+	Cases	% HIV+	Cases
*Age Group*	*∗*		*∗*		*∗*		*∗*	
15–24	4.3	3842	1.1	3089	4.9	4504	2.1	3450
25–34	10.3	3035	7.0	2248	10.3	3331	6.3	2493
35–44	9.3	1741	9.3	1603	11.5	2247	11.1	2006
45–59	6.6	1448	6.6	1295	8.5	1760	8.1	1574
*Wealth/poverty Status *	*ns*		*ns*		*ns*		*ns*	
Lowest	6.4	1767	4.3	1360	8.1	2278	6.9	1677
Second	7.4	1948	5.4	1583	9.2	2358	5.1	1933
Middle	7.5	2070	5.2	1688	7.4	2303	6.2	1901
Fourth	7.3	2145	5.7	1692	7.4	2407	6.5	2012
Highest	7.9	2236	5.2	1914	8.9	2495	6.0	2001
*Educational Attainment*	*∗*		*∗*		*∗*		*∗*	
No education	5.6	2531	7.2	741	8.6	1923	7.5	597
Incomplete primary	7.6	4657	4.8	3862	8.7	5481	6.6	4055
Complete primary	9.9	1100	6.2	1178	9.4	1441	7.0	1390
Incomplete secondary	7.3	1579	4.8	1919	6.7	2334	5.2	2493
Complete sec & higher	8.6	279	4.4	524	5.4	662	4.6	989
*Area of Residence *	*∗*		*∗*		*ns*		*∗*	
Rural	6.4	8672	4.9	7087	7.6	9407	6.1	7711
Urban	12.6	1493	7.0	1151	10.8	2436	6.3	1813
*Current Marital Status *	*∗*		*∗*		*∗*		*∗*	
Never been in union	2.8	2084	0.8	2915	2.0	3204	3.9	2604
Married/living together	5.7	6428	6.6	4624	7.4	5710	7.0	7430
Widowed	24.0	803	27.0	141	22.7	75	24.0	692
Divorced/separated	14.0	850	11.1	557	15.0	534	16.4	1117
*Sex of Household Head *	*∗*		*∗*		*∗*		*∗*	
Male headed household	5.4	6864	5.6	7073	6.1	7264	6.4	7992
Female headed household	11.4	3301	2.9	1165	11.6	4578	4.7	1532
Total	7.3	10165	5.2	8238	8.2	11842	6.1	9524

^*∗*^Statistically significant at 5% significance level, chi-square *P* < 0.05, ns: not significant at 5% level, *P* > 0.05.

**Table 2:** Odds ratios (OR) of being HIV positive for women and men, Uganda AIS, 2004-2005 and 2011 (*n* = 39,766).

Parameters	Model 1		Model 3		Model 4	
	OR	95% CI	OR	95% CI	OR	95% CI
*Fixed effects*						
Constant	0.06	[0.06–0.08]^*∗*^	0.02	[0.02–0.03]^*∗*^	0.01	[0.00–0.01]^*∗*^
*Model 1: Socio-economic factors*						
*Wealth status (Ref: Lowest)*						
Second	1.04	[0.90–1.20]	1.07	[0.93–1.24]	1.04	[0.90–1.20]
Middle	1.00	[0.86–1.15]	1.06	[0.91–1.23]	1.02	[0.88–1.19]
Fourth	1.02	[0.88–1.18]	1.09	[0.93–1.27]	1.05	[0.90–1.22]
Highest	1.05	[0.90–1.22]	1.20	[1.02–1.40]^*∗*^	1.15	[0.98–1.35]
*Education (Ref: No)*						
Incomplete primary	0.91	[0.80–1.04]	1.23	[1.08–1.41]^*∗*^	1.12	[0.98–1.28]
Complete primary	1.00	[0.86–1.18]	1.32	[1.12–1.56]^*∗*^	1.17	[0.99–1.39]
Incomplete secondary	0.67	[0.57–0.78]^*∗*^	1.10	[0.93–1.30]	0.95	[0.80–1.13]
Complete secondary & higher	0.54	[0.43–0.68]^*∗*^	0.74	[0.58–0.94]^*∗*^	0.63	[0.49–0.81]^*∗*^
Missing	1.20	[0.34–4.23]	1.70	[0.47–6.10]	1.58	[0.44–5.65]
*Model 2: Other socio-economic factors *						
*Place of residence (Ref: Urban)*						
Rural			0.52	[0.44–0.60]^*∗*^	0.60	[0.51–0.70]^*∗*^
*Ethnicity (Ref: Baganda)*						
Banyankole/Bakiga			1.18	[0.99–1.40]	1.43	[1.21–1.70]^*∗*^
Iteso/Karimojong			0.75	[0.60–0.93]^*∗*^	0.86	[0.69–1.07]
Lubgbara/Madi			0.48	[0.36–0.62]^*∗*^	0.61	[0.47–0.80]^*∗*^
Basoga			0.79	[0.63–0.98]^*∗*^	0.76	[0.62–0.95]^*∗*^
Langi/Acholi			1.22	[0.99–1.50]	1.42	[1.16–1.75]^*∗*^
Bagisu/Sabiny			0.80	[0.61–1.05]	0.73	[0.56–0.95]^*∗*^
Alur/Japadhola			0.79	[0.61–1.03]	0.83	[0.64–1.08]
Banyoro/Batoro			1.36	[1.11–1.67]^*∗*^	1.33	[1.08–1.64]^*∗*^
All others			0.87	[0.72–1.04]	0.95	[0.80–1.14]
*Time in years (Ref: 2004-05)*						
2011			1.23	[1.09–1.39]^*∗*^	1.23	[1.09–1.38]^*∗*^
*Model 3: Socio-demographic factors *						
*Age of respondent (Ref: 45–59 years)*						
15–24 years			0.82	[0.69–0.97]^*∗*^	1.08	[0.91–1.29]
25–34 years			1.53	[1.34–1.75]^*∗*^	1.71	[1.49–1.96]^*∗*^
35–44 years			1.69	[1.48–1.93]^*∗*^	1.77	[1.54–2.03]^*∗*^
*Marital status (Ref: Never been married)*						
Married/living together			2.32	[1.94–2.77]^*∗*^	2.22	[1.81–2.73]^*∗*^
Widowed			11.12	[8.88–13.93]^*∗*^	7.94	[6.24–10.11]^*∗*^
Divorced/separated			5.22	[4.28–6.36]^*∗*^	3.60	[2.90–4.47]^*∗*^
*Sex of respondent (Ref: Men)*						
Women			1.13	[1.03–1.24]^*∗*^	1.72	[1.54–1.91]^*∗*^
*Model 4: Sexual practices*						
*Drunk with alcohol (Ref: No)*						
Drunk					1.25	[1.12–1.40]^*∗*^
Not applicable					0.27	[0.02–4.84]
*Condom use during risky sex (Ref: No)*						
Used condom					2.31	[2.00–2.67]^*∗*^
Not applicable					5.83	[0.33–103.37]
*Multiple sexual partners (Ref: 1 partner)*						
2–4 partners					2.13	[1.86–2.44]^*∗*^
>4 partners					3.87	[3.29–4.54]^*∗*^
Not applicable					0.82	[0.58–1.14]
*HIV/AIDS knowledge (Ref: No knowledge)*						
Lowest knowledge					1.13	[0.80–1.59]
Medium knowledge					1.12	[0.82–1.52]
Highest knowledge					1.39	[1.03–1.89]^*∗*^
*Random effects *						
Cluster variance	0.566	0.049^*∗*^	0.363	0.039^*∗*^	0.313	0.036^*∗*^
Clusters	887		887		887	
Individual	39766		39766		39766	

OR: odds ratios; 95% CI: confidence intervals.  ^*∗*^Statistical significance at 5%, *P* < 0.05.

**Table 3:** Odds ratios (OR) of being HIV positive for women and men, Uganda AIS, 2004-2005 and 2011 (*n* = 39,766) controlling for age in single-year dummy variables.

Parameter	Model 1		Model 2		Model 3		Model 4	
	OR	95% CI	OR	95% CI	OR	95% CI	OR	95% CI
*Fixed Effect*								
Constant	0.06	[0.06–0.08]^*∗*^	0.13	[0.10–0.16]^*∗*^	0.01	[0.01–0.02]^*∗*^	0.00	[0.00–0.01]^*∗*^
*Model 1: SES factors*								
*Wealth (Ref: Lowest)*								
Second	1.04	[0.90–1.20]	1.03	[0.89–1.18]	1.07	[0.92–1.23]	1.03	[0.89–1.19]
Middle	1.00	[0.86–1.15]	0.98	[0.85–1.13]	1.06	[0.91–1.23]	1.02	[0.88–1.18]
Fourth	1.02	[0.88–1.18]	1.00	[0.86–1.16]	1.08	[0.93–1.26]	1.04	[0.89–1.21]
Highest	1.05	[0.90–1.22]	1.06	[0.91–1.24]	1.18	[1.00–1.38]	1.13	[0.96–1.33]
*Education (Ref: No education)*								
Incomplete primary	0.91	[0.80–1.04]	0.87	[0.76–0.99]^*∗*^	1.23	[1.08–1.41]^*∗*^	1.12	[0.97–1.28]
Complete primary	1.00	[0.86–1.18]	0.92	[0.78–1.08]	1.31	[1.11–1.54]^*∗*^	1.16	[0.98–1.37]
Incomplete secondary	0.67	[0.57–0.78]^*∗*^	0.58	[0.49–0.68]^*∗*^	1.09	[0.92–1.29]	0.95	[0.80–1.12]
Complete secondary & higher	0.54	[0.43–0.68]^*∗*^	0.43	[0.34–0.55]^*∗*^	0.69	[0.54–0.89]^*∗*^	0.61	[0.48–0.79]^*∗*^
Missing	1.20	[0.34–4.23]	1.22	[0.35–4.27]	1.83	[0.51–6.60]	1.71	[0.47–6.15]
*Model 2: Other SES factors *								
*Place of residence (Ref: Urban)*								
Rural			0.51	[0.43–0.59]^*∗*^	0.52	[0.45–0.61]^*∗*^	0.60	[0.52–0.70]^*∗*^
*Ethnicity (Ref: Baganda)*								
Banyankole/Bakiga			1.13	[0.95–1.34]	1.17	[0.98–1.39]	1.42	[1.19–1.68]^*∗*^
Iteso/Karimojong			0.67	[0.54–0.84]^*∗*^	0.74	[0.59–0.93]^*∗*^	0.85	[0.68–1.06]
Lubgbara/Madi			0.45	[0.35–0.60]^*∗*^	0.47	[0.36–0.62]^*∗*^	0.60	[0.46–0.79]^*∗*^
Basoga			0.77	[0.62–0.95]^*∗*^	0.78	[0.63–0.97]^*∗*^	0.76	[0.61–0.94]^*∗*^
Langi/Acholi			1.13	[0.92–1.39]	1.22	[0.99–1.50]	1.42	[1.16–1.75]^*∗*^
Bagisu/Sabiny			0.75	[0.57–0.98]^*∗*^	0.80	[0.61–1.04]	0.73	[0.56–0.95]^*∗*^
Alur/Japadhola			0.72	[0.56–0.94]^*∗*^	0.80	[0.62–1.05]	0.84	[0.64–1.09]
Banyoro/Batoro			1.28	[1.04–1.58]	1.35	[1.09–1.66]^*∗*^	1.33	[1.08–1.63]^*∗*^
All others			0.82	[0.69–0.98]^*∗*^	0.86	[0.72–1.04]	0.95	[0.79–1.14]
*Time in years (Ref: 2005-05)*								
UG6			1.22	[1.08–1.38]^*∗*^	1.23	[1.08–1.39]^*∗*^	1.22	[1.08–1.37]^*∗*^
*Model 3: socio-demographics *								
*Age of respondent (Ref: 15 years)*								
Age 16					0.94	[0.50–1.75]	0.86	[0.46–1.61]
Age 17					1.04	[0.57–1.90]	0.88	[0.48–1.64]
Age 18					1.66	[0.97–2.84]	1.24	[0.70–2.18]
Age 19					1.85	[1.07–3.19]^*∗*^	1.33	[0.74–2.37]
Age 20					1.89	[1.11–3.22]^*∗*^	1.27	[0.72–2.24]
Age 21					2.95	[1.74–5.01]^*∗*^	1.86	[1.05–3.30]^*∗*^
Age 22					2.74	[1.63–4.63]^*∗*^	1.75	[1.00–3.08]^*∗*^
Age 23					2.84	[1.67–4.83]^*∗*^	1.75	[0.98–3.11]
Age 24					2.51	[1.48–4.26]^*∗*^	1.51	[0.85–2.67]
Age 25					2.90	[1.73–4.86]^*∗*^	1.73	[0.99–3.02]
Age 26					3.36	[1.97–5.71]^*∗*^	1.98	[1.12–3.51]^*∗*^
Age 27					3.33	[1.96–5.65]^*∗*^	1.89	[1.07–3.35]^*∗*^
Age 28					3.81	[2.28–6.35]^*∗*^	2.24	[1.29–3.90]^*∗*^
Age 29					5.01	[2.98–8.42]^*∗*^	2.80	[1.60–4.92]^*∗*^
Age 30					4.41	[2.66–7.29]^*∗*^	2.55	[1.48–4.41]^*∗*^
Age 31					4.60	[2.72–7.77]^*∗*^	2.45	[1.39–4.33]^*∗*^
Age 32					4.73	[2.83–7.92]^*∗*^	2.67	[1.53–4.66]^*∗*^
Age 33					5.91	[3.49–9.99]^*∗*^	3.20	[1.81–5.64]^*∗*^
Age 34					4.96	[2.91–8.43]^*∗*^	2.76	[1.56–4.89]^*∗*^
Age 35					4.90	[2.94–8.17]^*∗*^	2.64	[1.52–4.60]^*∗*^
Age 36					4.29	[2.51–7.33]^*∗*^	2.33	[1.31–4.15]^*∗*^
Age 37					5.37	[3.15–9.15]^*∗*^	2.93	[1.65–5.22]^*∗*^
Age 38					4.29	[2.54–7.25]^*∗*^	2.25	[1.27–3.96]^*∗*^
Age 39					5.37	[3.13–9.23]^*∗*^	2.84	[1.59–5.08]^*∗*^
Age 40					3.96	[2.35–6.67]^*∗*^	2.17	[1.24–3.82]^*∗*^
Age 41					5.26	[3.05–9.07]^*∗*^	2.79	[1.55–5.01]^*∗*^
Age 42					4.21	[2.45–7.21]^*∗*^	2.14	[1.20–3.83]^*∗*^
Age 43					4.64	[2.58–8.34]^*∗*^	2.38	[1.28–4.46]^*∗*^
Age 44					4.57	[2.58–8.10]^*∗*^	2.38	[1.29–4.38]^*∗*^
Age 45					3.66	[2.13–6.27]^*∗*^	1.82	[1.02–3.25]^*∗*^
Age 46					3.26	[1.80–5.89]^*∗*^	1.64	[0.88–3.08]
Age 47					3.57	[1.97–6.45]^*∗*^	1.84	[0.98–3.46]
Age 48					3.18	[1.80–5.63]^*∗*^	1.60	[0.87–2.96]
Age 49					4.62	[2.57–8.30]^*∗*^	2.34	[1.25–4.37]^*∗*^
Age 50					1.69	[0.92–3.11]	0.85	[0.44–1.62]
Age 51					2.87	[1.52–5.39]^*∗*^	1.44	[0.74–2.81]
Age 52					3.03	[1.67–5.51]^*∗*^	1.54	[0.82–2.92]
Age 53					2.75	[1.39–5.47]^*∗*^	1.41	[0.69–2.90]
Age 54					2.07	[1.06–4.04]^*∗*^	1.11	[0.55–2.24]
Age 55					1.50	[0.72–3.11]	0.77	[0.36–1.65]
Age 56					1.67	[0.82–3.43]	0.81	[0.38–1.72]
Age 57					2.20	[1.06–4.58]^*∗*^	1.13	[0.53–2.44]
Age 58					1.93	[0.95–3.92]	0.99	[0.47–2.09]
Age 59					1.51	[0.66–3.47]	0.74	[0.31–1.75]
*Marital status (Ref: Never)*								
Married/living together					1.65	[1.36–2.00]^*∗*^	2.00	[1.61–2.47]^*∗*^
Widowed					8.12	[6.39–10.31]^*∗*^	7.18	[5.61–9.19]^*∗*^
Divorced/separated					3.74	[3.02–4.63]^*∗*^	3.22	[2.58–4.02]^*∗*^
*Sex of respondent (Ref: Men)*								
Women					1.16	[1.05–1.27]^*∗*^	1.73	[1.55–1.93]^*∗*^
*Model 4: Sexual Practices *								
*Drunk alcohol (Ref: No)*								
Drunk							1.23	[1.10–1.37]^*∗*^
Not applicable							0.26	[0.01–4.89]
*Used condom (Ref: No)*								
Used condom							2.34	[2.02–2.71]^*∗*^
Not applicable							6.22	[0.34–115.28]
*Sexual partners (Ref: 1 partner)*								
2–4 partners							2.08	[1.82–2.38]^*∗*^
>4 partners							3.76	[3.19–4.42]^*∗*^
Not applicable							0.99	[0.68–1.44]
*HIV/AIDS Knowledge (Ref: No)*								
Lowest knowledge							1.12	[0.79–1.58]
Medium knowledge							1.11	[0.82–1.52]
Highest knowledge							1.38	[1.02–1.87]^*∗*^
*Random Effects*								
Cluster variance	0.566	0.049^*∗*^	0.385	0.039^*∗*^	0.365	0.039^*∗*^	0.318	0.036^*∗*^
Units: Cluster	887		887		887		887	
Units: Individual	39766		39766		39766		39766	
Estimation:	IGLS (MQL1)		IGLS (PQL2)		IGLS (PQL2)		IGLS (PQL2)	

OR: odds ratios; 95% CI: confidence intervals.  ^*∗*^Statistical significance at 5% level, *P* < 0.05.

**Table 4:** Odds ratios (OR) of HIV prevalence in rural and urban areas, UAIS, 2004-2005 and 2011.

Parameter	Rural				Urban			
	Model 1		Model 2		Model 1		Model 2	
	OR	95% CI	OR	95% CI	OR	95% CI	OR	95% CI
*Fixed effects*								
Constant	0.05	[0.04–0.06]^*∗*^	0.00	[0.00-0.00]^*∗*^	0.21	[0.15–0.29]^*∗*^	0.02	[0.01–0.03]^*∗*^
*Model 1: Socio-economic factors*								
*Wealth status (Ref: Lowest quintile)*								
Second	1.04	[0.88–1.23]	1.06	[0.89–1.26]	1.18	[0.92–1.52]	1.13	[0.87–1.46]
Middle	1.01	[0.85–1.20]	1.05	[0.88–1.25]	1.15	[0.88–1.50]	1.18	[0.89–1.55]
Fourth	1.13	[0.95–1.34]	1.17	[0.98–1.39]	0.88	[0.66–1.18]	0.89	[0.66–1.21]
Highest	1.17	[0.98–1.40]	1.23	[1.02–1.47]^*∗*^	0.87	[0.64–1.19]	0.99	[0.71–1.36]
*Education (Ref: No education)*								
Incomplete primary	0.91	[0.79–1.04]	1.15	[0.99–1.33]	0.67	[0.48–0.92]^*∗*^	0.93	[0.66–1.32]
Complete primary	1.04	[0.87–1.24]	1.30	[1.08–1.57]^*∗*^	0.54	[0.37–0.78]^*∗*^	0.73	[0.49–1.07]
Incomplete secondary	0.68	[0.57–0.82]^*∗*^	1.07	[0.88–1.30]	0.35	[0.25–0.49]^*∗*^	0.64	[0.45–0.93]^*∗*^
Complete secondary & higher	0.63	[0.45–0.87]^*∗*^	0.83	[0.59–1.17]	0.25	[0.17–0.38]^*∗*^	0.43	[0.28–0.67]^*∗*^
Missing	1.05	[0.23–4.84]	1.21	[0.26–5.71]	1.27	[0.12–13.37]	2.49	[0.21–29.13]
*Model 2: Socio-demographic factors*								
*Age of respondents (Ref: 45–59 years)*								
15–24 years			1.12	[0.91–1.37]			0.89	[0.63–1.27]
25–34 years			1.80	[1.54–2.12]^*∗*^			1.46	[1.08–1.96]^*∗*^
35–44 years			1.90	[1.62–2.22]^*∗*^			1.53	[1.13–2.07]^*∗*^
*Current marital status (Ref: Never been in union) *								
Married/living together			2.28	[1.75–2.97]^*∗*^			2.03	[1.45–2.83]^*∗*^
Widowed			8.75	[6.47–11.83]^*∗*^			6.14	[3.97–9.49]^*∗*^
Divorced/separated			3.92	[2.97–5.16]^*∗*^			2.97	[2.09–4.21]^*∗*^
*Sex of respondent (Ref: Men)*								
Women			1.61	[1.42–1.82]^*∗*^			2.13	[1.71–2.64]^*∗*^
*Time in years (Ref: 2004-05)*								
2011			1.33	[1.14–1.54]^*∗*^			0.97	[0.78–1.21]
*Random effects*								
Cluster variance	0.584	0.058^*∗*^	0.487	0.053^*∗*^	0.175	0.052^*∗*^	0.162	0.052^*∗*^
Clusters	719		719		168		168	
Individuals	32506		32506		7260		7260	

OR: odds ratios; 95% CI: confidence intervals. ^*∗*^Statistical significance at 5% level, *P* < 0.05.

**Table 5:** Odds ratios of HIV prevalence showing significant interactions with place of residence, UAIS, 2004-2005 and 2011.

Parameter	Model 1		Model 2		Model 3	
	OR	95% CI	OR	95% CI	OR	95% CI
*Fixed effects*						
Constant	0.09	[0.08–0.10]^*∗*^	0.20	[0.13–0.29]^*∗*^	0.01	[0.00–0.03]^*∗*^
*Model 1: Place of residence *						
*Place of residence (Ref: Urban)*						
Rural	0.58	[0.50–0.68]^*∗*^	0.45	[0.28–0.71]^*∗*^	0.30	[0.10–0.90]^*∗*^
*Model 2: Socio-economic factors *						
*Wealth status (Ref: Lowest)*						
Second			1.14	[0.88–1.47]	1.20	[0.91–1.57]
Middle			1.12	[0.85–1.46]	1.27	[0.95–1.69]
Fourth			0.86	[0.64–1.16]	0.98	[0.71–1.34]
Highest			0.85	[0.62–1.17]	1.05	[0.75–1.47]
Second. Rural			0.88	[0.65–1.18]	0.84	[0.61–1.16]
Middle. Rural			0.85	[0.62–1.17]	0.77	[0.55–1.07]
Fourth. Rural			1.22	[0.87–1.70]	1.10	[0.77–1.58]
Highest. Rural			1.28	[0.89–1.83]	1.08	[0.73–1.59]
*Education (Ref: No education)*						
Incomplete primary			0.68	[0.49–0.93]^*∗*^	0.95	[0.67–1.35]
Complete primary			0.54	[0.38–0.78]^*∗*^	0.75	[0.50–1.11]
Incomplete secondary			0.36	[0.26–0.50]^*∗*^	0.66	[0.45–0.95]^*∗*^
Complete secondary & higher			0.26	[0.17–0.38]^*∗*^	0.42	[0.27–0.65]^*∗*^
Missing			1.11	[0.10–12.23]	3.04	[0.27–34.38]
Incomplete primary. Rural			1.30	[0.92–1.84]	1.15	[0.79–1.70]
Complete primary. Rural			1.83	[1.22–2.73]^*∗*^	1.64	[1.06–2.55]^*∗*^
Incomplete secondary. Rural			1.85	[1.27–2.69]^*∗*^	1.55	[1.02–2.37]^*∗*^
Complete secondary & higher. Rural			2.41	[1.46–3.99]^*∗*^	1.91	[1.10–3.33]^*∗*^
Missing. Rural			1.00	[0.06–16.57]	0.43	[0.02–7.63]
*Ethnicity (Ref: Baganda)*						
Banyankole/Bakiga			1.43	[1.07–1.91]^*∗*^	1.64	[1.21–2.24]^*∗*^
Iteso/Karimojong			1.41	[0.94–2.12]	1.74	[1.14–2.67]^*∗*^
Lubgbara/Madi			1.17	[0.75–1.82]	1.53	[0.94–2.48]
Basoga			1.11	[0.79–1.57]	1.14	[0.79–1.64]
Langi/Acholi			1.52	[1.02–2.27]^*∗*^	2.03	[1.33–3.08]^*∗*^
Bagisu/Sabiny			1.19	[0.71–1.98]	1.02	[0.59–1.77]
Alur/Japadhola			0.79	[0.46–1.35]	1.00	[0.57–1.74]
Banyoro/Batoro			1.60	[1.12–2.27]^*∗*^	1.62	[1.11–2.37]^*∗*^
All others			0.94	[0.68–1.29]	1.16	[0.83–1.62]
Banyankole/Bakiga. Rural			0.62	[0.44–0.89]^*∗*^	0.71	[0.49–1.04]
Iteso/Karimojong. Rural			0.35	[0.22–0.56]^*∗*^	0.36	[0.22–0.59]^*∗*^
Lubgbara/Madi. Rural			0.26	[0.15–0.44]^*∗*^	0.26	[0.14–0.46]^*∗*^
Basoga.Rural			0.51	[0.33–0.79]^*∗*^	0.50	[0.32–0.78]^*∗*^
Langi/Acholi. Rural			0.60	[0.38–0.96]^*∗*^	0.55	[0.34–0.90]^*∗*^
Bagisu/Sabiny. Rural			0.48	[0.27–0.87]^*∗*^	0.56	[0.30–1.05]
Alur/Japadhola. Rural			0.80	[0.44–1.48]	0.68	[0.36–1.29]
Banyoro/Batoro. Rural			0.64	[0.42–1.00]	0.66	[0.42–1.04]
All others. Rural			0.74	[0.51–1.09]	0.67	[0.45–1.00]
*Time in years (Ref: 2004-05)*						
UG6 (2011)			0.96	[0.75–1.23]	1.00	[0.78–1.29]
UG6.Rural			1.29	[0.98–1.71]	1.26	[0.95–1.67]
*Model 3: Socio-demographic factors *						
*Age of respondent (45–59 years)*						
15*–*24 years					0.89	[0.62–1.28]
25*–*34 years					1.42	[1.05–1.92]^*∗*^
35*–*44 years					1.50	[1.10–2.04]^*∗*^
15*–*24 years. Rural					1.27	[0.84–1.91]
25*–*34 years. Rural					1.27	[0.90–1.78]
35*–*44 years. Rural					1.24	[0.88–1.76]
*Sex of respondent (Ref: Men)*						
Women					2.18	[1.75–2.71]^*∗*^
Women. Rural					0.74	[0.57–0.95]^*∗*^
*Marital status (Ref: Never been married)*						
Married/living together					2.02	[1.44–2.84]^*∗*^
Widowed					6.22	[3.99–9.68]^*∗*^
Divorced/separated					2.99	[2.09–4.27]^*∗*^
Married/living together. Rural					1.14	[0.75–1.75]
Widowed. Rural					1.36	[0.80–2.32]
Divorced/separated. Rural					1.29	[0.82–2.02]
*Drunk with alcohol before risky sex (Ref: No)*						
Drunk					1.54	[1.22–1.95]^*∗*^
Not applicable					0.69	[0.03–14.80]
Drunk. Rural					0.78	[0.60–1.02]
Not applicable. Rural					0.32	[0.00–24.69]
*Condom use during risky sex (Ref: No)*						
Used condom					1.91	[1.48–2.47]^*∗*^
Not applicable					1.99	[0.09–42.92]
Used condom. Rural					1.34	[0.98–1.83]
Not applicable. Rural					3.78	[0.05–291.30]
*Multiple sexual partners (Ref: 1 sex partner)*						
2–4 partners					1.97	[1.47–2.64]^*∗*^
>4 partners					3.55	[2.54–4.95]^*∗*^
Not applicable					0.50	[0.25–1.02]
2–4 partners.Rural					1.09	[0.78–1.51]
>4 partners. Rural					1.10	[0.75–1.61]
Not applicable. Rural					1.89	[0.84–4.25]
*AIDS knowledge (Ref: No knowledge)*						
Lowest knowledge					0.66	[0.29–1.52]
Medium knowledge					0.77	[0.37–1.61]
Highest knowledge					0.89	[0.43–1.83]
Lowest knowledge. Rural					1.82	[0.73–4.54]
Medium knowledge. Rural					1.47	[0.65–3.31]
Highest knowledge. Rural					1.61	[0.73–3.57]
*Random effects*						
Constant	0.478	0.044^*∗*^	0.363	0.035^*∗*^	0.293	0.035^*∗*^
Cluster	887		887		887	
Individual	39766		39766		39766	

OR: odds ratios; 95% CI: confidence intervals.  ^*∗*^Statistical significance at 5% level, *P* < 0.05.

## References

[1] Fortson J. G. (2008). The gradient in sub-saharan Africa: socioeconomic status and HIV/AIDS. *Demography*.

[2] Parkhurst J. O. (2010). Understanding the correlations between wealth, poverty and human immunodeficiency virus infection in African countries. *Bulletin of the World Health Organization*.

[3] Fox A. M. (2012). The HIV-poverty thesis RE-examined: Poverty, wealth or inequality as a social determinant of hiv infection in sub-Saharan Africa?. *Journal of Biosocial Science*.

[4] Gillespie S., Greener R., Whiteside A., Whitworth J. (2007). Investigating the empirical evidence for understanding vulnerability and the associations between poverty, HIV infection and AIDS impact. *AIDS*.

[5] Msisha W. M., Kapiga S. H., Earls F., Subramanian S. V. (2008). Socioeconomic status and HIV seroprevalence in Tanzania: a counterintuitive relationship. *International Journal of Epidemiology*.

[6] Durevall D., Lindskog A. (2012). Economic Inequality and HIV in Malawi. *World Development*.

[7] Fotso J.-C., Kuate-Defo B. (2005). Measuring socioeconomic status in health research in developing countries: should we be focusing on households, communities or both?. *Social Indicators Research*.

[8] Whiteside A. (2002). Poverty and HIV/AIDS in Africa. *Third World Quarterly*.

[9] Gupta N., Mahy M. (2003). Sexual initiation among adolescent girls and boys: trends and differentials in sub-saharan Africa. *Archives of Sexual Behavior*.

[10] Glynn J. R., Carael M., Buvé A. (2004). Does increased general schooling protect against HIV infection? A study in four African cities. *Tropical Medicine & International Health*.

[11] Meekers D., Ahmed G. (2000). Contemporary patterns of adolescent sexuality in urban Botswana. *Journal of Biosocial Science*.

[12] Dinkelman T., Lam D., Leibbrandt M. (2007). Household and community income, economic shocks and risky sexual behavior of young adults: Evidence from the Cape Area Panel Study 2002 and 2005. *AIDS*.

[13] Marteleto L., Lam D., Ranchhod V. (2008). Sexual behavior, pregnancy, and schooling among young people in Urban South Africa. *Studies in Family Planning*.

[14] Magadi M. A. (2011). Understanding the gender disparity in HIV infection across countries in sub-Saharan Africa: evidence from the demographic and Health Surveys. *Sociology of Health and Illness*.

[15] de Walque D. (2007). How does the impact of an HIV/AIDS information campaign vary with educational attainment? Evidence from rural Uganda. *Journal of Development Economics*.

[16] Zuilkowski S. S., Jukes M. C. H. (2012). The impact of education on sexual behavior in sub-Saharan Africa: a review of the evidence. *AIDS Care*.

[17] Bradley H., Bedada A., Brahmbhatt H., Kidanu A., Gillespie D., Tsui A. (2007). Educational attainment and HIV status among Ethiopian voluntary counseling and testing clients. *AIDS and Behavior*.

[18] Messina J. P., Emch M., Muwonga J. (2010). Spatial and socio-behavioral patterns of HIV prevalence in the Democratic Republic of Congo. *Social Science & Medicine*.

[19] J. Madise N., Ziraba A. K., Inungu J. (2012). Are slum dwellers at heightened risk of HIV infection than other urban residents? Evidence from population-based HIV prevalence surveys in Kenya. *Health & Place*.

[20] Bloom S. S., Urassa M., Isingo R., Ng'weshemi J., Boerma J. T. (2002). Community effects on the risk of HIV infection in rural Tanzania. *Sexually Transmitted Infections*.

[21] Hillemeier M. M., Lynch J., Harper S., Casper M. (2003). Measuring contextual characteristics for community health. *Health Services Research*.

[22] Stephenson R. (2009). Community factors shaping HIV-related stigma among young people in three African countries. *AIDS Care Psychological and Socio-medical Aspects of AIDS/HIV*.

[23] Tiruneh F. N., Chuang K.-Y., Ntenda P. A. M., Chuang Y.-C. (2017). Individual-level and community-level determinants of cervical cancer screening among Kenyan women: A multilevel analysis of a Nationwide survey. *BMC Women's Health*.

[24] Clarke P., Crawford C., Steele F., Vignoles A. F. The choice between fixed and random effects models: some considerations for educational research.

[25] Clarke P., Crawford C., Steele F., Vignoles A. (2015). Revisiting fixed- and random-effects models: some considerations for policy-relevant education research. *Education Economics*.

[26] Link B. G., Phelan J. (1995). Social conditions as fundamental causes of disease. *Journal of Health and Social Behavior*.

[27] Tuller D. M., Bangsberg D. R., Senkungu J., Ware N. C., Emenyonu N., Weiser S. D. (2010). Transportation costs impede sustained adherence and access to HAART in a clinic population in Southwestern Uganda: a qualitative study. *AIDS and Behavior*.

[28] Duff P., Kipp W., Wild T. C., Rubaale T., Okech-Ojony J. (2010). Barriers to accessing highly active antiretroviral therapy by HIV-positive women attending an antenatal clinic in a regional hospital in western Uganda. *Journal of the International AIDS Society*.

[29] Parkhurst J. O. (2002). The Ugandan success story? Evidence and claims of HIV-1 prevention. *The Lancet*.

[30] Joint United Nations Programme on HIV/AIDS (UNAIDS) (2014). *Global Report: UNAIDS Report on the Global AIDS Epidemic 2013*.

[31] Granados J. M. S., Amador J. T. R., De Miguel S. F. (2003). Impact of highly active antiretroviral therapy on the morbidity and mortality in Spanish human immunodeficiency virus-infected children. *The Pediatric Infectious Disease Journal*.

[32] Quinn T. C. (2008). HIV epidemiology and the effects of antiviral therapy on long-term consequences. *AIDS*.

[33] Poorolajal J., Hooshmand E., Mahjub H., Esmailnasab N., Jenabi E. (2016). Survival rate of AIDS disease and mortality in HIV-infected patients: a meta-analysis. *Public Health*.

[34] Wang Y., Jin F., Wang Q., Suo Z. (2017). Long-Term Survival of AIDS Patients Treated with only Traditional Chinese Medicine. *AIDS Research and Human Retroviruses*.

[35] World Bank (WB) The World Bank Annual Report.

[36] National Planning Authority [Uganda] National development report 2010-2011 financial year.

[37] Uganda Bureau of Statistics (UBOS) and Macro International Inc Uganda Demographic and Health Survey, 2006.

[38] Mishra V., Vaessen M., Boerma J. T. (2006). HIV testing in national population-based surveys: experience from the demographic and health surveys. *Bulletin of the World Health Organization*.

[39] Ministry of Health [Uganda] and ICF International (2012). *Uganda AIDS Indicator Survey*.

[40] Uthman O. A., Moradi T., Lawoko S. (2009). The independent contribution of individual-, neighbourhood-, and country-level socioeconomic position on attitudes towards intimate partner violence against women in sub-Saharan Africa: A multilevel model of direct and moderating effects. *Social Science & Medicine*.

[41] Rutstein S. O., Johnson K., Macro O. (2004). The DHS wealth index , ORC Macro. *MEASURE DHS*.

[42] Rutstein S., Staveteig S. (2014). Making the demographic and health surveys wealth index comparable.

[43] Rodrigo C., Rajapakse S. (2010). HIV, poverty and women. *International Health*.

[44] Magadi M. A. (2013). The disproportionate high risk of HIV infection among the urban poor in sub-Saharan Africa. *AIDS and Behavior*.

[45] Magadi M. A. (2016). Understanding the urban–rural disparity in HIV and poverty nexus: the case of Kenya. *Journal of Public Health*.

[46] Rasbash J., Steele F., Browne W., Goldstein H. (2009). *A User’s Guide to MLwiN, v2. 10*.

[47] Obalenskaya P. (2012). *Attitudes towards family and marriage in time and context: using two British birth cohorts for comparison [Ph.D. in Sociology]*.

[48] Asiedu C., Asiedu E., Owusu F. (2012). The socio-economic determinants of HIV/AIDS infection rates in Lesotho, Malawi, Swaziland and Zimbabwe. *Development Policy Review*.

[49] Tarling R. Statistical modelling for social researchers: principles and practice, Taylor and Francis.

[50] Mishra V., Assche S. B.-V., Greener R. (2007). HIV infection does not disproportionately affect the poorer in sub-Saharan Africa. *AIDS*.

[51] Mavedzenge S. N., Olson R., Doyle A. M., Changalucha J., Ross D. A. (2011). The epidemiology of HIV among young people in sub-Saharan Africa: know your local epidemic and its implications for prevention. *Journal of Adolescent Health*.

[52] Magnani R. J., Karim A. M., Weiss L. A., Bond K. C., Lemba M., Morgan G. T. (2002). Reproductive health risk and protective factors among youth in Lusaka, Zambia. *Journal of Adolescent Health*.

[53] Barnighausen T., Hosegood V., Timaeus I. M., Newell M. L. (2007). The socioeconomic determinants of HIV incidence: evidence from a longitudinal, population-based study in rural South Africa. *AIDS*.

[54] Kelly M. J. What HIV/AIDS Can Do to Education, and what Education Can Do to HIV/AIDS, UN AIDS.

[55] Feldacker C., Ennett S. T., Speizer I. (2011). It's not just who you are but where you live: an exploration of community influences on individual HIV status in rural Malawi. *Social Science & Medicine*.

[56] Sumra S., Mugo J. Are our children learning? Assessment of learning outcomes among children in Tanzania, Kenya and Uganda.

[57] Jones S., Schipper Y., Ruto S., Rajani R. (2014). Can your child read and count? Measuring learning outcomes in East Africa. *Journal of African Economies*.

[58] Edmonds A., Yotebieng M., Lusiama J. (2011). The effect of highly active antiretroviral therapy on the survival of HIV-infected children in a resource-deprived setting: a cohort study. *PLoS Medicine*.

[59] Igulot P.

